# The Hygienic Treatment of Pulmonary Consumption

**Published:** 1856-10

**Authors:** Benjamin W. Richardson

**Affiliations:** Physician to the Royal Infirmary for Diseases of the Chest.


### ON THE HYGIENIC TREATMENT OF PULMONARY CONSUMPTION.

By BENJAMIN W. RICHARDSON, M.D., Physician to the Royal Infirmary for Diseases of the Chest.

### PRELIMINARY CONSIDERATIONS.

THE progress of hygienic medicine in the last few years is the medical fact of the present age, and the fact that will stand out in boldest relief when the history of this period shall be written by some future Esculapian scholar.

But, rapid and effective as this progress has been, the principles of hygiene are yet but in their infancy. We have learned to appreciate the true value of hygienic principles in the prevention of various diseases, especially those of the epidemic type; and the medical profession, throwing aside all selfish recollections, has been the first to teach the practice of these principles, and to prove their force and vitality. The next step in the way of advancement is to demonstrate that the same principles are as useful and as necessary in the treatment of actual disease as they are in prevention. In this field of labour ground is indeed broken. Our physicians are trusting in part to the ventilation of the hospital ward, and to a careful regimen, for the cure of many of their patients; while Stromeyer and others are showing absolutely that the one and true remedy for the typhus-stricken victim is pure air in abundance.

A great advantage in the hygienic treatment in disease is, that it does not, or at least need not, interfere with sound and experience-proved modes of treatment of a medicinal kind. An ague patient is benefited by hygienic measures, but this is no reason why he should not at the same time be subjected to the well known curative effects of cinchona. An anæmic child is made to inhale pure air, and to exercise its limbs, but this is no reason why it should not also be medicinally plied with steel. In fact, in the hands of the scientific physician, there is always a consistent plan for combining the medicinal and hygienic systems. He sees that the two systems are one; he sees further that the mere medicinal plan without the hygienic is in all cases imperfect, and in some cases worse than imperfect. Will all the medicines in the Pharmacopœia cure small-pox in a patient shut out from the air, and breathing steadily his own poison emanations? The dream of such a possibility is past now.

The practical details of hygienic medicine in relation to the treatment of disease have, however, yet to be wrought out more fully. This will be sure work, but slow. Necessarily slow, because it is hard to give up old friendships in dogmatism; and to effect a cure in a sick man by fresh air alone, or diet, is infinitely less satisfactory to the public, than to assume to effect the same cure by a bread pill. I know some earnest men in the profession who are afraid to abandon the routine of pills, draughts, lotions, and what not, because such abandonment knocks head against the foibles of those who seek to be cured by such means, and who will recognise no other means. Nor is there want of argument in this course, though its morality may be open to criticism. A dispensary patient came to me lately, to be cured of a headache. I saw what seemed to be the cause, and without prescribing on the letter, gave in words what promised to be the remedy. The patient came no more; but I asked after her casually one day of a friend who brought her to see me. "Oh!" was the reply, "she has no faith in you; you only told her to sleep in a room with a chimney in it, and to sleep alone; so now she is getting cooling medicine from a druggist, and some days she is worse, and some days she is better." Always worse, I take it, after the cooling medicine. It would be easy to multiply these illustrations, but this one is a fair representation of others.

It is vain, it is sticking in the slough of hopelessness, to pander to these popular imbecilities; for though they must die out, and, indeed, are dying out daily, they will go the sooner if they are effectually damped, and if something real and common sense is put in their place. *Scientiæ mutantur, et nos mutamur in illis.* There is a time when medicines are invaluable; but if faith in medicines is to be retained, the times for their administration, as well as their selection, must be learned by knowledge, not by routine; and must be dictated by the circumstances of the case, not by the caprice of the patient. The executive of science must be independent, if it would keep in the path of truth and advancement.

In such progress as has been made in the science of treatment by medicines, it has been found useful to take up certain particular diseases, and to observe in them, individually, the effects of particular remedies. This rule will apply with equal force in considering and investigating hygienic modes of treatment. Each practitioner should, as his opportunities permit, observe as carefully the effects of his hygienic commands, as he does those of the medicines he may prescribe. He should compare also the one mode with the other, and calculate in each case their relative advantages.

In this way he will have the advantage of detecting with greater accuracy the pure effects of medicines themselves; seeing that the action of medicines is greatly modified by the external conditions to which he who takes them is subjected.

Convinced of the importance of the above considerations, I have made it my business for some time past to mark out a series of hygienic rules for the treatment of consumptives; and as I have been happily favoured by the best and widest opportunities of carrying out these rules in practice, and as the results have been most satisfactory, I lay the views, given in succeeding chapters, respectfully and briefly before the profession and the public, but without making the proposition of anything like a specific cure for the disease in question.

The idea of a hygienic code for consumptives is by no means new, but it has as yet been limited and incomplete. On the diet of consumptives volumes have been written, and specific diets have been invented as abundantly as have specific pills and plasters. Out-door exercise also, the necessity of which it will be my task earnestly to enforce, has been often urged, and some conscientious men have earned for themselves not a little disrepute by the pertinacity with which they have pressed their views on this point on the attention of the public. An American physician, Dr. Parrish, in a number of the *North American Medical and Surgical Journal* for 1830, wrote thus:—

"Vigorous exercises, and a free exposure to air, are by far the most efficient remedies in pulmonary consumption. It is not, however, that kind of exercise usually prescribed for invalids—an occasional walk or ride in pleasant weather, with strict confinement in the intervals—from which much good is to be expected. Daily and long continued riding on horseback or in a carriage is, perhaps, the best mode of exercise; but where this cannot be commanded, unremitting exertion of almost any kind in the open air, amounting even to labour, will be found highly beneficial. Nor should the weather be scrupulously studied. Though I would not advise a consumptive patient to expose himself recklessly to the severest inclemencies of the weather, I would, nevertheless, warn him against allowing the dread of taking cold to confine him on every occasion when the temperature may be low, or the skies overcast.

"I may be told that the patient is often too feeble to be able to bear exertion; but except in the last stage, where every remedy must prove unavailing, I believe there are few who cannot use exercise out of doors ; and it sometimes happens that those who are exceedingly debilitated, find, upon making the trial, that their strength is increased by the effort, and that the more they exert themselves the better able they are to support the exertion."

M. Salvadori, of Trent, and a Mr. May espoused a similar view even long previous to the time of Dr. Parrish. These gentlemen, Salvadori and May, proposed and carried out, for consumptives, the plan of supplying them freely with the most nutritious foods, such as beef and wine, and of subjecting them also to vigorous exercises, such as climbing mountains and taking prolonged walks. Salvadori trusted to this alone, and ignored medicines. Mr. May used bark, opiates, and emetics, and conjoined a swinging gymnastic exercise to his treatment.

Dr. Rush gave an opinion that exercise in the cold air was useful in hæmoptysis, and commends the hardship of active military service as the most effectual remedy in many cases of confirmed consumption.

Recently Dr. Jackson, another American physician, has taught the same doctrine in his work entitled *Letters to a Young Physician*, and has gone further than Dr. Parrish towards forming an extended hygienic system of treatment. He has given instructions on diet and clothing, but his great argument, like that of his predecessor, is in favour of exercise, and of free exposure at all times to a pure air, in consumptive cases.

I must not omit to refer also to the opinions of Dr. M'Cormac, which practically run in the same direction, but which are connected with a theory held by him as to the origin of tubercle. It is apart from my present purpose to discuss the question of the origin of tubercle, and I therefore need only refer to the fact, that pure air in abundance is, in the opinion of Dr. M'Cormac, the essential preventive against the commencement of consumption, and the most essential remedy when the disease has made its appearance.

### OUTLINE OF A HYGIENIC CODE FOR THE TREATMENT OF CONSUMPTIVES.

In giving the following rules I presuppose their general applicability to cases of consumption in all stages of the disease : in the premonitory stage ; in the stage when the tubercular deposition is apparent ; and in the next stage, when the local mischief is much further advanced. In the last stage even, though hope is lost, many of the rules may still be rigidly followed out with advantage, for by them the course of the disease is smoothed, and sometimes life is prolonged. In like manner, the rules are generally applicable to those who by hereditary taint are as yet but predisposed to the disease.

Rule i.—*A supply of pure air for respiration is the first indication in the treatment of the consumptive patient.* In all cases of consumption, the attention of the physician should be at once directed to the quality of the air breathed by the patient. It may seem dogmatical, but it is true, that in an atmosphere containing one per cent. of the carbonic acid of the breath, with the natural but as yet undetermined amount of ammonia evolved with the carbonic acid, in such an atmosphere a consumptive patient, though in the earliest stage of the disease, cannot possibly recover under any form of medicinal treatment; while in those predisposed to the disease, the inhalation of such an atmosphere, even at intervals, will aid materially in inducing the first symptoms of the disease. In saying one per cent. of carbonic acid, I have taken a high figure, because it is known that in health the respiration of such an atmosphere for a long time is hurtful. How much more so in consumption, where the patient, by reason of the imperfect play of the lungs, is already taking in too little air!

In large cities, and even in small towns, it is next to impossible to get a constantly pure air in inhabited houses, for houses are built according to false notions of comfort. "What a nice cozy room," is a common expression applied innocently to every place where the greatest care has been taken to make an air vault, without a "draught," and all ready for being charged with invisible impurities.

In a cozy room the consumptive is bound never to live, nor in any room indeed for great lengths of time. So long as he is able to be out of doors, he is in his best and safest home. In the fields, on the hills, wherever the fresh air vivifies, where plants look most vigorous, and animals frisk about in the joy of health, there will the consumptive draw in his choicest medicine, there dissolve and throw off most freely the germs of his disease, and there repair most easily the tissues he has lost.

The inclemencies of the weather may temporarily, it is true, prevent the patient from his out-door existence. But even these inclemencies are not so much to be dreaded as confinement in a house. I had occasion, some time since, repeatedly to remark that if, from a few days rain, the consumptives under my care were confined to their homes, instead of being able to take the daily out-door breathing always prescribed, under such circumstances the aggravation of symptoms was always marked and universal. The appetite fell off, the debility became greater, the mind was less buoyant, the local mischief increased. The patients, too, previously accustomed to a full dose of the air food, were not ignorant of the cause of these changes, for reduction in air is felt as quickly as reduction in common diet. Seeing these evils, then, I have lately thrown off the alarm about bad weather, and have ordered every patient to seize on an inclement day each gleam of sunshine, for the purpose of getting out for a breath of fresh air. The result of this practice has been most gratifying in all cases where the courage of the patient has admitted of its application.

Dr. Jackson, in speaking of out-door life, in much the same terms as the above, dwells very properly on the necessity of securing for this plan the confidence of the patient. The treatment "should not be done rashly, but boldly." If possible, "the patient should be made to have faith in it; for without this he is not likely to pursue it as far as he can, and then he will not derive from it all the benefit which it can afford." This is the fact; but the difficulty is at once got over if, under favourable conditions, the invalid can be induced to try the measure for a few days. Once tried, there is no fear, in the majority of cases, of its being given up, except in instances where the disease is too far advanced, or where, from the poverty of the patient, the pursuit of a sedentary occupation must needs be followed, even to the last days of existence; for the benefit derived from the proceeding is so plain, the debility is so much better borne, the relish for food is so much more markedly felt, the nights are passed with so much less of restlessness and cough, and with such an increase of sleep, that the sufferer soon instinctively feels the value of his instructions, and follows them out even more punctually than those which relate to the taking of medicines.

As much of the day, then, as is possible should be spent by the consumptive in the open air, and in places where the air is least impeded and least corrupted. When he is compelled to keep the house, the necessary precautions must again be taken for procuring a free admission of the atmosphere. No cozy room with a temperature at 70°, with every crevice closed, and with an atmosphere in a dead calm and laden with impurities, should be permitted. But the temperature should be from 55° to 65° Fahr.; the fire, if there is one, should be in an open grate; and by perforated panes in the windows, and a free chimney vent secured by an Arnott valve, the freest possible current of air should be kept circulating through the room. If the patient is cold, let him approach the fire, but let him not labour under the popular and fatal error, that the way to obtain animal warmth is to shut out the air and roast the body. The heat of the body is made in the body itself, by virtue mainly of the oxygen supplied in the air; and as the body absorbs external heat with great difficulty, it would be as wise to attempt to give warmth by fires, hot bottles, and hot air, to a man who is not inhaling a due amount of oxygen, as to attempt the same process on a marble statue. In a word, external heat is useful only in preventing the too rapid radiation of animal heat from the surface of the animal body. Alone, it cannot supply heat; but when a wholesome air is inspired, it can secure the retention of the heat that is manufactured in the animal furnace.

I spoke a moment ago of the open fire-grate. This is an essential for the room of the consumptive. Stoves of all kinds, heated pipes, and, in a word, every mode of supplying artificial warmth, except that obtained by the radiation from an open fire, is, according to the facts which I have been able to collect, injurious. It is injurious, because by such means the air is made too dry, an objection much less applicable to the open fire. If compelled to live in a room heated by a stove or by hot water pipes, or if the air in a room heated by an open fire be too dry, as may occur during north-east winds, the consumptive patient should meet the difficulty by allowing the steam from boiling water to be diffused through the apartment. I have known a patient to be kept awake with constant dry cough during the whole night from what seemed, in great part, the dryness of the atmosphere, and have been able to afford relief by the simple suggestion named above.

But the evil effects arising from the common closed stove are as nothing compared with the system of heating an apartment by hot air passing into the room from an iron flue. It is a fortunate fact that this monstrous mode is now fast going into disuse, for a second time in this country. The air thus heated bears with it minute irritating particles, which to healthy lungs are hurtful, and to phthisical lungs fatal.

The symptom which I have most commonly seen elicited in the phthisical, by the inhalation of an unnaturally dry air, is hæmoptysis, a symptom brought on possibly by the constant cough which the dry air excites. This effect, in a minor degree, will, in fact, appear in some cases without any actual deposition of tubercular matter under the influence of the cause just described. A gentleman whom I knew, and whose lungs were free from tubercle and other organic disorder, was constantly annoyed and troubled with slight attacks of hacking cough and blood-spitting. He was at a loss to account for the cause. At last he detected that the attacks always commenced when he was at work in his study. With the idea of being very warm and comfortable, and ignorant of the nature of animal heat, he had introduced into a small room a large Burton's stove. To a stranger entering that room when the stove was in action, and the doors and windows snugly closed, the heat and dryness of the atmosphere would have been at once oppressive; but he, a close student, and constantly occupying the room under such conditions, had become accustomed to it as regards external sensation, but caught the mischief effectually in the chest. The cause of the symptoms being explained, the stove was abandoned, and the open fire-grate was again resorted to: the cough and blood spitting at once disappeared without the administration of any medicine. A few weeks afterwards, thinking that the stove and the cough might only stand in the position of coincidenccs, our student resumed the use of the stove: and what is more, resumed also, as an effect, the cough and the blood expectoration. This time he became assured that the stove and cough stood in the relation of cause and effect. The cause was once more removed, and ever since he has remained free of the effect.

The temperature of the air in the room of the consumptive should range from 55° to 65° Fahr., and he himself should learn to observe by the thermometer that he is living in an air of this degree of warmth. Bennett's shilling thermometers answer for this purpose admirably, and come within the means of the poorest patient.

I must say a word about schoolrooms in this place. If any father or mother have a child of consumptive tendency, I beg them to send him to no school until they have personally inspected the schoolroom, in regard to its ventilation and to the mode in which it is warmed.

The worst constructed stoves, the worst plans of ventilation, are, I regret to say, to be found in these public rooms, even in those of the best class; and boys are not uncommonly punished for being stupid, when they are really in a semitorpid state from the effects of one of the most active gaseous narcotics—carbonic acid. If a child of consumptive birth be carefully brought up, with strict regard to sound physiological rules, there is always a chance of carrying him fairly into manhood, and beyond the period at which tubercle so commonly presents itself. But if such a child be sent from home to spend six or eight hours a-day amongst a troop of other children, in an unventilated room, dry heated by iron stoves, that child has no chance; his disease is being drilled into him, *pari passu*, with his learning, and the end is forthcoming.

In order to ascertain the degree of moisture in the air, Dr. Arnott recommends the use of the hygrometer. This would be most advantageous, and the sensations of a consumptive patient would soon inform him what degree of moisture was comfortable and proper. But there is yet a desideratum in practical hygiene, to which the Rev. C. Girdlestone drew attention in the JOURNAL OF PUBLIC HEALTH for March 1855; viz., an instrument for indicating at all times the amount of carbonic acid gas present in the air of any apartment, and as simply constructed as the barometer or thermometer.

I have occasionally heard phthisical patients complain of the use of gas in the rooms where they are confined. Such complaints, however, have usually come from patients confined in workshops where the number of burners is very great, and where there is almost always some accidental escape of gas.

In private houses such objections are avoidable; but as the inhalation of coal gas is injurious even in small quantities, and as the products of the combustion of such gas are also hurtful, the necessity of a free ventilation in rooms where it is burned and in which consumptives are lodged, is the more urgent.

The care that should be taken to secure a good air in the living rooms of the phthisical invalid, must extend with equal care to the sleeping apartment. This rule should always obtain when possible; *never permit one room to perform the two offices of bedroom and living room.* The bedroom should be large, unencumbered by needless furniture, and thoroughly ventilated. If the temperature of the air without is not below 60° Fahr., the windows of the room should be boldly set open, and be kept open all night. If they are to be closed of necessity, a free chimney draught must be procured, and an Arnott's valve is always an advantage. In the absence of this, a bent tube may be used, as described in another page of this work. The bed should be free of curtains, but a single screen may be placed so as to ward off any direct draught from the door or window. Warmth of body is best secured by woollen bedclothes; but if the temperature of the air is below 60°, it will with advantage be raised to that pitch by a fire in the open grate. Gas should on no pretence be burned through the night in this bedroom, and as few other lights as possible, for the patient requires all the air that is to be had, and must not be carelessly robbed of it. Above all things, the consumptive person should be the sole occupant of his own bed and bedroom. To place such an one for several hours close to another person, however healthy, is injurious to both, but especially to the sick. No ties of relationship, and no mistaken kindness, should cause this rule of isolation ever to be broken.

It has been stated already that the room of the sufferer should be large. It should include, whenever practicable, at least 1,000 cubic feet of breathing space, under all plans of ventilation. If more space can be had, all the better. If less only is obtainable, then the ventilation must be the more carefully attended to.

When the patient has left room in the morning, and he should do so early, the windows and doors should be set open, and a current of air be allowed to flow through during the whole of the day. If the air of the apartment be at a temperature below 60° Fahr., or loaded with moisture, the fire should be lighted before bedtime. In thus preparing a bedroom for the reception of the sick, I have known nurses, either in ignorance or in idleness, take up a chaffer of lighted coke, or a warming-pan full of live coals, and set it in the centre of the room to effect the "airing" process. This act is nothing less than a systematic diffusion of coke poison.

*Use of respirators.*—Consumptive patients frequently ask, especially in winter time, the value of what are called respirators; and I have known some poor people to purchase things of this description at what was to them considerable cost. The use of mufflers, which are, in fact, respirators, has been known for ages; and Dr. Hales, more than a century ago, recommended a scientifically made muffler for persons obliged to enter into places where noxious gases were given off. Dr. Beddoes too, as Dr. Arnott shows, pointed out, in the year 1802, that a few folds of gauze held over the mouth and nose made the air warm and moist for respiration, and that such mufflers were, therefore, useful to consumptive and asthmatic persons. The object of the muffler or respirator is this; it retains the heat thrown out in the expired air, and gives up this heat to the cold air that enters in inspiration. In cold dry weather the muffler is very useful, and should be worn by all phthisical patients when out of doors; but when the air is moist and cold it sometimes is complained of, as embarrassing the respiration. It should then be thrown aside. Any patient may easily make one of these mufflers for himself, for the cost of a few pence, out of a piece of fine wire gauze, cut oval so as to cover the mouth and nose, and fixed in the centre of a handkerchief, so that it may be tied on like an ordinary comforter, with the gauze in the centre for breathing through.

Before leaving the subject of pure air as a remedy for the consumptive, I regret to be obliged to offer an opinion which is, I know, exceptional, and which is therefore given with the firmness of a conscientious conviction, but with the respect due to the opinions of the majority. I am about to speak of the confinement of consumptives in hospitals. That a vast deal of good is, or may be, done at these institutions by the treatment prescribed by the physicians who attend at them, and whose lives are devoted to the study of the disease, there cannot be a doubt. But that it is either physiological, or sound practical treatment, to receive into these buildings consumptive patients, is an assumption I must most earnestly dispute. I know the excellent spirit in which institutions of this kind are founded. I am fully aware of the care that is bestowed on the inmates; of the attempts that are made to introduce every hygienic improvement; of the order and cleanliness that prevail; of the kindness of the attendants; of the excellence of the diet roll; and of the skill of the physicians. With all this, it is to me as clear as crystal, that to bring phthisical patients into such institutions is a great charitable mistake. The very care, and waiting servant attention, that is paid to such of the invalids as are in the first and second stages of the disease, is a cruel kindness. The remedy for them is to encourage and urge them to assist themselves, and to exert themselves. Moreover, no kind of hygienic system, carried on in a large building filled with inmates, can make the air of that building in any way equal to the outer air, which it is so necessary that the consumptive person should breathe. Twenty patients, lying in one hospital ward, will throw off per minute into the air of the ward at least three and a half cubic feet of expired and impure gases, rendered in the phthisical the more impure by the pathological condition of the lungs. But the impure air thus exhaled vitiates by its diffusion twenty times its own volume of pure air ; so that, in fact, in a ward with twenty patients, there are not less than seventy cubic feet of air spoiled per minute, and rendered unfit for the purposes of life. It may be granted that during the day, when the wards are less full, and many windows are open, and the movements of the inmates are active, the expired air may be fairly disposed of. But take a winter night of twelve hours ; consider that in this period of time the twenty patients would, if they exhaled even naturally, vitiate fifty thousand four hundred cubic feet of air, which ought to be removed, and to be replaced by two thousand five hundred and twenty cubic feet of *pure* air for the use of respiration ; and then reflect whether it is probable that such a ward can remain during the whole night uncontaminated. For, granting to the twenty patients a breathing space of twenty-six thousand cubic feet, and even then it would require that the whole of the air in that space should be removed and replaced by fresh air fully twice in the one night. Against this, possibly, the artificial ventilating argumentists will urge that such a feat of ventilation is nothing at all, not worth considerng, so easy to be done. M. Grouvelle would probably undertake to effect such interchange eight times in the night, or more ; and if he undertook to do it eighty times, and did not succeed in doing it once, it might be difficult to prove the fact against him. But if he would take a strip of paper prepared for ozone, place it in a ward, however artificially ventilated, and place another similar paper in the open air adjoining the ward, it is a mistake if he should not find that there was a striking difference in the process of oxidation in the two localities ; and that the great life supporter, oxygen, was in a condition to play a very much more active part in its out-door than in its in-door work.

The misfortune of a great hospital, with all its rooms communicating indirectly with each other, is, that the ventilation is always uncertain. There is, in fact, no properly ventilated space except the great vault of heaven, and no true ventilating power except in the combinations of atmospheric pressure, wind movements, and the force of diffusion.

If special hospitals for consumptives are to be had, they should be as little colonies, situated far away from the thickly populated abodes of men, and so arranged that each patient should have a distinct dwelling place for himself. They should be provided with pleasure grounds of great extent, in which the patients who could walk about should pass every possible hour in the day; and with glass covered walks overhead, where they could breathe open air, and yet be dry, even if rain were falling. Very expensive such an establishment would be, there is no doubt; but it would, I take it, be infinitely more practically advantageous to treat ten patients in this manner, than ten tens in a confined brick and mortar box, through which of necessity some amount of invisible impurity, some trace of transparent poison cloud, is constantly floating.

The strongest argument in favour of consumption hospitals is, that they receive those members of the community who could not at their own homes afford the same advantages as are supplied to them in the charity. Against this it is to be urged that the patients taken into the consumption hospitals are *not*, in this country at least, in any way to be considered as the representatives of the most needy and destitute sections of the community. These latter go to their last homes in the workhouse, or in their own poverty stricken dwellings. The classes that fill the hospitals are often many grades above destitution; and are sometimes comparatively wealthy. They have access to a governor who gives them an admission letter, and they leave their own medical adviser to enter the hospital, not because they cannot find the means to live at home and be treated at home, but because, catching at every new suggestion offered to them, they set their hearts on getting into the hospital, as though it were a certain haven of rescue. In this scramble after admission some of course succeed; they leave their homes, they enter the hospital, and there the greater proportion of them either die or return back to their friends nearer death than before. A few recover or are relieved; but whether the same result would have occurred, if they had been subjected to the same medical and general treatment out of the hospital? is a question which may be left very safely answered in the affirmative.

Rule ii.—*Active exercise is an essential element in the treatment of Consumptives.* The conditions for obtaining a due supply of air imply in some measure the necessity for exercise. But there are varieties of exercise. We have seen that Drs. Rush, Jackson, and Parrish are in favour of riding on horseback, but this is a thing not practically to be carried out in the majority of cases, and, as I think, not absolutely necessary. Walking is the true natural exercise, and the best, for it brings into movement every part of the body more or less, and, leading to brisker circulation in every part, causes a more active nutrition generally. The extent to which exercise should be carried will vary with the stage of the disease, and temporary accidents may for the moment stop it altogether, such, for instance, as an attack of hæmoptysis. But when exercise is advisable, the general rule is to recommend that it be carried out systematically, cautiously, and courageously, and that each exercise should be continued until a gentle feeling of fatigue is felt through the whole muscular system. Violent and unequal exertion of the upper muscles of the body is unadvisable. When restored from the fatigue of one exertion, another should be undertaken, and during the day this cannot be too often repeated. If the day be wet, then the exercise should be effected by walking in a large room, or by engaging in some game, such as skittles, billiards, or tennis.

If, in his waking hours, the consumptive patient can keep himself occupied pretty freely in muscular labour, he secures the best sudorific for his sleeping hours that can possibly be supplied; for as the cause of force is always expended in producing motion or action, so, to use the words of Dr. Metcalfe, "the proximate cause of sleep is an expenditure of the substance and vital energy of the brain, nerves, and voluntary muscles, beyond what they receive when awake; and the specific office of sleep is the restoration of what has been wasted by exercise." Cough is very much less frequent in the course of the night in him who has been subjected to exercise in the day; while sleep, when it falls, is more profound, more prolonged, and more refreshing.

In summer time, when the temperature of the day is high, the morning and the evening time are the best adapted for the periods of out-door exertion. In the other seasons, midday is preferable, as a general rule.

I have sometimes been asked whether what are called gymnastic exercises are commendable in consumptive cases, and whether swinging is good. My idea on these points is that, in swinging, a person is much more usefully exercised when throwing the swing for his associates' pleasure, than in being himself swung. There is, in fact, but little faith to be placed in so-called scientific gymnastics. Anything that a man invents to overtop or compete with nature must needs be paltry. Brisk natural movement of the limbs is all that the consumptive requires. He need not go out of his way after a sham, in the shape of a shampooer; chopping wood is a good gymnastic feat, and playing at skittles is perfect in its way.

The value of exercise is threefold. First, it checks waste of muscular structures, for muscles left inactive undergo a consumption, without any necessity for lung disorder. Secondly, it diverts the blood from the lungs, causes a more brisk circulation through them, and a more free distribution through the system at large. Thirdly, it induces a more free respiration; more oxygen is taken into the lungs, the body is restored to its vital purposes more surely, and, just in proportion as this restoration is effected, so is the restoration of disordered function and of disorganised tissue.

In the performance of muscular exercise let the consumptive never encumber himself, or check the free movements of his body by strappings, loads of clothes, or carrying of weights, and the like. These are but tasks; they lead to unequal exertion in special sets of muscles, and such inequality of expenditure is that which is to be avoided. The treatment of consumption in a hospital is objectionable, again, in regard to exercise. Of what use to the consumptive is an acre or two of airing ground confined at the back of his hospital? Let him be certain that where the gardener cannot make roses bloom, and peach trees blossom, no doctor can give to the anæmic cheek a permanent colour, to a lost function its uses, or to an impoverished body its once healthy power.

A last consideration on the value of muscular exercise is, that it is eminently useful in keeping the respiratory muscles in a state of active nutrition. For, if to the loss of capaciousness in the lungs to receive air, there is added a daily increasing failure in the muscles by which the acts of inspiration and expiration are carried on, it is clear that a double evil is at work. Now this double evil is most actively presented in consumption. As the respiratory muscles, together with the other muscles, lose their tone, so do the general symptoms of exhaustion increase in severity; sometimes without very marked change in the pathological condition of the lungs. As a sequence, day by day, as the nutrition of these muscles decreases, and as they fail in tonic contractile power, they gain in excitability; so that the irregular spasmodic contractions to which they are subjected in the act of coughing are produced by the merest excitement, and the cough is more frequent as it becomes more feeble.

Rule III.—*A uniform climate is an important element in the treatment of Consumptives.* Consumptive patients are constantly asking questions as to the value of a change of climate. The poorest applicants for relief are anxious on this point, and are often ready at once to contemplate emigration, if the merest hope is given to them that such a course would prove beneficial. Several patients under my care have thus, while in the first stage of the disease, gone away, some to Western Australia, some to the South of Ireland, two to the Cape of Good Hope, and one to Valparaiso. Their fate I do not know. Patients sometimes have friends living in distant parts of the world, to whom they would like to go if such change of climate is recommended. In these cases I look at a map of the district, and obtain some geographical information regarding it before giving an opinion. Mr. Keith Johnston's *Physical Atlas,* and his paper on the "Geographical Distribution of Disease throughout the Globe",* are documents of great value in this respect, since they give the physical characters of each country, and a history of its most prevalent diseases. In considering climate, the fact should be remembered that the main point to be obtained is to select such a part of the earth's surface as presents the nearest approach to an equality of temperature. Different writers of eminence have given the most contrary opinions on climate and consumption. Some have recommended a warm climate, others the polar regions. Both parties have spoken from experience, and they are, in some measure, both right; for a climate equally cold, and a climate equally hot, are each much more favourable than one in which there are constant variations, and where the thermometer in the course of the year dances about from many degrees below freezing point, up to 100° or more. Speaking of the mortality of consumption in 153,098 deaths between the years 1841 and 1851, the Irish Census Commissioners thus observe:—

"As might naturally be expected, the seasons exercised a very marked influence upon the deaths from consumption. During the mild months of autumn, succeeding the warm season of summer, the deaths attributed to consumption amounted to only 23,010; with the cold of winter the mortality from this cause increased, so as to present a return of 38,956; but with the harsh trying weather of spring it rose to 51,334, and in summer fell again to 39,798."†

This statement represents a very important truth. It is certainly best for the patient, if the temperature, while equal, is also temperate; but a mean temperature of 35° on

* For this paper, see JOURNAL OF PUBLIC HEALTH for July 1856.

† Census of Ireland for the year 1851. Report on Tables of Deaths, p. 448.

one side, or 75° on the other, is preferable to one varying constantly, to-day at 60° Fahr., to-morrow at 40°, and a few days later at 80°.

In taking charge of a large number of consumptive patients attending for relief at an institution, it is a remarkable and highly instructive task to observe the influence of climatic changes in the symptoms of the disease. As each day comes for attendance at the Royal Infirmary for Diseases of the Chest, I can predict, almost with absolute certainty, what is the history I am to hear from the consumptives who are coming before me. If for some days there has been an uniformity of temperature, and the weather has been mild and dry, so that an airing each day out of doors has been effected, the visit is quite a cheery one; all seem better; the medicines are said to agree. The cough is less troublesome, the body is warmer, and hope, throwing an inward sunshine, lights up each face with brightness and activity. In frosty days, too, when the air is dry and the temperature continues even, the symptoms are often equally favourable; but during periods, so common in this country in the spring and in the beginning of winter, when the atmospheric variations are sudden, marked, and often repeated in the course of a few weeks, the general aspect of affairs is widely different. I have heard on these occasions almost every patient complaining; the symptoms are all exaggerated, the mind discontented. There is a general request for a change in the medicine. Something is asked for that will soothe, for the nights are passed indifferently. It is useless to comply always with these demands, since the exaggerated train of complaints has a general and common cause; but now and then the modification of symptoms is so great as to call for a modification of treatment.

During these seasons of variation, deaths from consumption are most prevalent. Thus an equable temperature is of great moment, and should always be sought after by the phthisical sufferer. If he cannot remove from his own locality, and if the variations in it are considerable, he must meet them by the best precautions at command. In-doors it is not difficult to sustain a pretty even temperature, varying from 50° to 60° Fahr. Out of doors, something must be done by attention to clothing, and by the use of the respirator. The most marked variations, however, occur in the night, and hence the importance of keeping up an equality of warmth in the bedroom, in the manner already described.

The reasons why consumptives feel the effects of climatic changes so much, are sufficiently obvious. The effects of such variations are felt, indeed, in the best health; for the body is in some measure both a barometer and a thermometer, at all events it is subject to the same influences, the lungs being in all cases the parts most affected. With the temperature moderately high and the air dry, the physiology of respiration is carried on easily and well. The amount of oxygen taken in is ample, the expiration of water, carbonic acid, and ammonia is free; the pulmonic circuit of the blood is unimpeded; the exhalation of water from the skin is unchecked; and the radiation of heat from the body is moderate. Let the atmospheric condition suddenly change for one in which the temperature is 35°, or less, and in which the air is charged with watery vapour; and the conditions of life are materially modified. The supply of oxygen taken into the lungs is less; the process of absorption of such oxygen by the blood is less; the products expired are less; the pulmonic circulation is impeded; the watery exhalation from the skin is in part suppressed; the radiation of heat from the body is much more rapid; and, as a result of all, the whole man, body and mind, is reduced in force and in vitality. This is the course of things in a healthy man during atmospheric variations. It is left with the reader to trace out the exaggerated evil of these changes in those who, at the most favourable times, are existing with the lungs reduced in capaciousness and the respiratory muscles in power.

I shall recommend no particular place as a resort for consumptives; for I wish not to enter into disputation on this point. But here is the formula for an hypothetical consumptive Atlantis. It should be near the sea coast, and sheltered from northerly winds; the soil should be dry; the drinking water pure; the mean temperature about 60°, with a range of not more than ten or fifteen degrees on either side. It is not easy to fix any degree of humidity; but extremes of dryness or of moisture are alike injurious. It is of importance in selecting a locality that the scenery should be enticing, so that the patient may be the more encouraged to spend his time out of doors in walking or riding exercise, and a town where the residences are isolated and scattered about, and where drainage and cleanliness are attended to, is much preferable to one where the houses are closely packed, however small its population may be.

In speaking thus of the value of an equal climate, I am guided entirely by the facts daily presented to me in relation to climatic variations on patients living in or near

London. Some authors, however, infer from mortality returns, gathered from various quarters of the world, that variations of climate do not materially affect the disease. The following are Mr. Keith Johnston's remarks.

"Tubercular consumption cannot be said to be a disease peculiar to any one portion of the globe, or to be dependent on climate in any appreciable degree, unless it can be shown that it does not prevail in the excessive climates of the north. It originates in all latitudes from the equator, where the mean temperature is 80°, with slight variations, to the higher portion of the temperate zone, where the mean temperature is 40°, with sudden and violent changes. The opinion long entertained, that it is peculiar to cold and humid climates, is founded in error. Far from this being the case, the tables of mortality of the army and navy of this and other countries, as well as those of the civil population, warrant the conclusion that consumption is more prevalent in tropical than in temperate countries. Consumption is rare in the Arctic regions, in Siberia, Iceland, the Faroe islands, the Orkneys, Shetlands, and Hebrides. And in confirmation of the opinion that it decreases with the decrease of temperature, Fuchs shows, from extensive data, that in northern Europe it is most prevalent at the level of the sea, and that it decreases with increase of elevation to a certain point. At Marseilles, on the seaboard, the mortality from this cause is 25 per cent.; at Oldenburg, 80 feet above the sea, it is 30 per cent.; at Hamburg, 48 feet above the sea, it is 23 per cent.; while at Eschwege, 496 feet above the sea, it is only 12; and at Brotterode, 1800 feet above the sea, 0·9 per cent. It is calculated that in the temperate zone, within which nearly all the civilised inhabitants of the globe are located, at least one-tenth of the population die of this malady. It is uniformly more fatal in cities than in the country: in England the excess in cities is equal to 25 per cent."

But the facts here related are not opposed to the rule of climatic uniformity when carefully weighed. On the contrary, they go with the rule; for as consumption is most rare in extreme northern climates, and at great elevations, so in these localities are variations of climate less marked. It remains yet for statistics to show whether in more favoured patches of earth, where with the same absence of climatic variations there is a more genial but temperate warmth, the disease is equally prominent and fatal.

The Reports of the Irish Census Commissioners, already noticed, add, however, more force to the rule I have laid down, than any facts as yet published. The mortality from consumption in the spring months, for ten years, is there shewn to be twenty-two thousand more than in any other season. Why? Not, it is clear enough, because the months of spring are hotter than those of winter, or colder than those of summer; but because, in this transition season, the variations of climate are more severely felt. It is "the peevish April day" that tells, in its numerous changes, its cold mists, its warm sun, its heavy showers, on the constitution of the consumptive man.

Rule iv.—*The dress of the consumptive patient should be adapted to equalise the temperature of the body.* Instinctive sensations both in health and disease naturally dictate the above rule. But it is too commonly the fact that these sometimes are disobeyed. Some persons think it a hardy, and therefore a beneficial plan to dress lightly in all weathers. Foolish mothers send out their children in midwinter with bare legs and chests; young ladies go to balls and evening parties with the upper part of their dresses open, to show off more effectually a finely chiselled throat and bosom. A lady hints to me that this is the custom of society, not the vanity of the sex. Admitted, madam, out of courtesy; but is society to have its victims from the innocent? It has enough, I take it, if it has them from the wicked only. Others go on a different tack; they must at all seasons be smothered up in flannels and outer dresses, layer upon layer, carrying in short as much cloth as they possibly can, like a fast sailing cutter. Such persons on both sides evidently misunderstand the uses of clothes, or think them only ornamental appendages. But clothes are useful, in a sanitary point of view, simply for equalising temperature, *i. e.*, for preventing more or less the escape of the animal heat as it is radiated from the external surface of the body. Heat is transmitted slowly through flannel, so flannel is warm. For this reason, some say that flannel should be worn in summer as well as in winter, because in winter it retains the animal heat, and in summer it prevents the external heat from oppressing the body. The last part of this argument is a mistake, as experience teaches. For the body does not get its heat from without but from within, and the course of the heat is always from within outwards into space. If, therefore, as occurs in summer, the body cannot barter its heat freely enough to the warm air which surrounds it, it becomes hot; but surely it is no good policy to prevent such radiation as would go on, by interposing a layer of flannel between the body and the air. A loose flannel outer dress may be passable in hot weather, because the air circulates freely beneath it; but a closely fitting flannel underdress is just as unnecessary in this case, as it is necessary in winter when the air robs the system of more heat than can be conveniently spared.

I speak here of the body in health. In the consumptive patient, the principle is modified. He, from the deficient play of his lungs, is virtually always living in winter; and you shall find him on the hottest days breathing with anxiety, and with his hands and brow cold as marble.

For the consumptive, therefore, flannel clothing is always required, and it should cover the whole of his body. The poorest man or woman may avail themselves of this, for it matters little what the outer garments are if the under ones are non-conductors of heat. The thickness of flannel must vary according to the sensations; as far as is possible, the feeling of absolute cold ought to be at all times prevented. The consumptive should sleep also in flannel; not in the dress worn during the day, but in a flannel gown. The shoes worn should be thick, whole, and comfortable. All sorts of absurdities in the way of hair skins, warm plaisters, and the like, placed specially on the chest, are useless; and the plaister is worse than useless, since it checks the function of the skin over a considerable surface, and is dirty.

A common practice in the selection of clothes is to imagine that the weight of a garment conveys an idea of its warmth-sustaining power. This is an absurd error; for what is warmer than a German coverlet, which is simply a silk bag half filled with down, and nearly as light as air? For the consumptive persons, this mistake about heavy clothing must be carefully avoided; they may safely trust to flannel, and may then walk out as warm as they can be made by clothing, without the risk of being wearied from the burthen on the back before they have got half a mile from home.

There is one modern article of male attire, on which a word of caution must be said, for its bad effects are unmistakeable. I must warn men in general, and consumptive men in particular, against wearing what are called waterproof India rubber coats. That these intolerable nuisances are very tempting there is no doubt; they are light; they are rainproof; and are they not reversible, two-faced, so that one may be transformed by them, in half a minute, from the similitude of a cabman into the representative of a very spruce gentleman? But let any one walk in one of these portable bathing machines, with the shiny side inwards, for an hour or so, and at a brisk pace; and, when he has finished his journey, let him look at the shiny side within, and feel the hygrometric state of his under coat; and if he does not find that there is such a thing as getting wet through without rain, and that anything preventing a drenching of the body from without is certain to check the exhalations from the skin, he must be very blind to the defects of the reversible. The healthy man may tolerate one of these garments; the consumptive, never. They load the under clothes with moisture; they give a cold envelope to the surface; they produce chill; and, by checking the cutaneous function, they throw a double amount of work on lungs, already failing under their ordinary duties.

Is it necessary to more than mention those abominations of female attire, corsets? I hope not. However, as some young ladies are still led to imprison themselves in them, it may be well to tell the mothers of such, that to screw up a consumptive child's chest with stays, is only equivalent to preventing the act of breathing by the mouth, because it is performed with difficulty by the nose.

Rule v.—*The hours of rest of the consumptive patient should extend from sunset to sunrise.* If exercise is important to the consumptive patient during the day, a due allowance of sleep is equally necessary during the night. The natural hours of sleep are from sunset to sunrise, and it is the business of the consumptive to make nature his oracle. Shakespeare has happily said that sleep is the "chief nourisher in life's feast", and Menander held that it was "a remedy for every curable disease". The great use of sleep truly is to renovate; for in this state the formative processes go on most actively. Metcalfe, to whom I before referred, has well defined the difference between exercise and sleep, by saying "that during exercise the expenditure of the body exceeds the income; whereas during sleep the income exceeds the expenditure."

It is obvious that to the consumptive man nothing can be more important than that his income should exceed his expenditure; and it is quite remarkable how much alleviated all the symptoms of consumption are when the balmy god is appealed to not in vain. The rule I have laid down regarding the hours for sleep is imperative for many reasons. First, because in all seasons the actual amount of rest required by the natural man is pointed out with the precision of an astronomical law by the course of the sun. In midwinter men require, for physiological reasons, more sleep than they do at midsummer, and just so much more as is indicated by the difference of night in these two periods. Observe how all animals, left to their own natural instincts, obey this law. Secondly, in our present artificial mode of life, we have to extend the day by the invention of artificial lights. But whenever a man shuts himself up in his closet, and makes a little sun out of his gas lamp or candle, he is feeding that lamp with a part of his own breathing store—the air around him. Worse still, the candle can, no more than the man, live alight without exhaling carbonic acid gas, and thus vitiating the atmosphere. A pound of oil burnt in a lamp produces, in burning, nearly three pounds; and every cubic foot of coal gas, rather more than a cubic foot of carbonic acid. The evil effects of carbonic acid on the lungs have been already described. Thirdly, as an artificial light is, by the mode in which it is produced, of necessity injurious, so, on the contrary, the pure sunlight is of the greatest worth in the acts of vitality. What sunlight does in a physiological way is undetermined; but its general influence has long been known and recognised. Plants banked up from the light become blanched, and human beings kept for a long time in dark abodes become the victims of anæmia and scrofula.

Thus, to fulfil the natural law regulating the times of sleep, to escape from the artificial light, and to obtain the advantage of all the sun-light that can be secured, the consumptive patient should make the sun his fellow workman.

During the act of sleep many physiological modifications occur, which it is important to notice. In the sleeping state the number of respirations are diminished and the circulation is more feeble; as a result, the temperature of the body is reduced. These facts supply two indications, viz., that a free supply of air must be given to the sleeping man, and that he must be well enclosed in woollen material, so as to husband his animal heat. The profuse perspirations, which form so marked a symptom in the phthisical generally, come on during a profound doze, and the patient wakes to find himself bathed in moisture. It always occurs to me, that this profuse action of the skin is but secondary and consequent to a diminished exhalation from the lungs. At all events, after having tried oil inunctions, sponging with acid solution, and the administration of various astringent remedies, with varying success, I have found no plan so efficient for preventing these perspirations as that of supplying a constant current of pure air. This system does not, of course, interfere with the application of other remedial measures, but it should stand foremost. Cough also, so common a disturber of the night's repose, is most effectually treated on the ventilation principle. For an impure air excites cough by its direct effect on the mucous surface of the air passages; and further, as before shown, when air laden with carbonic acid is inhaled, the chemical changes of respiration are checked, the pulmonic circuit is retarded, the heart becomes embarrassed, and congestion of the lungs is an inevitable result. This is another exciting cause of cough and expectoration.

Rule vi. *The occupation of the consumptive patient should be suspended if it is in-door or sedentary; but a certain amount of out-door occupation may be advantageous.* This rule is one which, in the majority of cases, is most difficult to carry out, though second to none in importance. There is, in a word, no exciting cause of consumption so general as an in-door occupation. I remarked some time ago that about two out of every three patients with consumption, who presented themselves before me at the Infirmary, were found on inquiry to be employed in some in-door business. This was confirmed accurately by reference to the Infirmary books, the figures of which have been very carefully analysed for me by Mr. Pring, a student and assistant at this institution.

Of late, the occupation of every patient applying for relief has been noted down; and since this plan was commenced, there have been at the Infirmary five hundred and fifteen cases of consumption under the treatment either of my colleagues, Drs. Davies and Powell, or under my own care. From the pains that are taken in diagnosis in each of these cases, they may be all received as representing real instances of the disease, in one or other of its stages. Out of these five hundred and fifteen cases then, not less than 68·34 per cent., or rather more than two-thirds, have been persons following in-door occupations. Possibly the per centage is even higher, for all who have called themselves labourers have been presumed to be out-door workers, although this may not have been always the fact, since many labourers in London are employed in vaults, in warehouses, and in gas-works. Among the in-door occupations which present the largest number of cases in this list, boot and shoemakers rank first; needlewomen second; watch and clock-makers third; domestic servants fourth; painters fifth; tailors sixth; printers, of whom the majority are compositors, seventh; bookbinders eighth; French polishers ninth; cigar-makers tenth; writers eleventh; smiths twelfth; tinmen thirteenth; and cabinet-makers fourteenth. There are altogether in the list one hundred and forty trades specified, but the above named fourteen yield rather more than forty-four and a half per cent. of the whole.

I am aware that five hundred and fifteen cases, however carefully selected, are a small number from which to draw any very large conclusion; and I regret that the table of occupations and diseases given by the Irish Census Commissioners is too general and vague, in its application of terms, to admit of its being used in this place. But of the fact of the great preponderance of consumptive cases amongst persons who live in a confined space, there can be no reasonable doubt.

In the case of parents having children of a consumptive tendency therefore, the greatest care should be taken to obtain for them out-door employment. But here a serious delusion commonly comes into play. If the child is weakly, the fond parent urges, that it is unfit for hard labour and for out-door vicissitudes; so it is sent to a tailor or shoemaker, to a clerk's office, a draper's shop, or to some occupation of an in-door character; by this grand, ignorant, and fatal mistake, it is added to the list of the two-thirds who swell the tables of consumption cases.

In many in-door occupations a double mischief is at work. The patient is confined in an impure air, and is made to inhale some foreign agent, present of necessity from the character of his work, and with which the air is charged. I cannot here enumerate the substances which the lungs are thus made to inhale; they are as various as trades themselves. Sand and glass, in the sand-paper manufactory; dusts and fluffs of different kinds in textile manufactories; acid vapours in dyeing establishments; naphtha and turpentine vapours in polishing and burnishing shops; these are but a few examples.

Whenever a consumptive patient following an in-door occupation comes under treatment, he or she must be made either to leave it or to modify it. Some occupations, such as cigar making, sand-paper making, and fur dying, are absolutely fatal, and it is hopeless to treat medicinally the patient who continues to follow them. But in other trades, where no mechanical mischief is being done to the lungs, and where the evils mainly are those of confinement in a room and want of exercise, very much can be done by ventilation, and by getting the sufferer to give up a portion of every day to a long walk in the open air.

Almost all occupations implying muscular exertion out of doors, without undue exposure to wet and damp, may often be pursued by the consumptive as long as possible, and with advantage. The pursuit of some occupations is better exercise than simple walking, since it keeps the mind occupied and in healthful tune.

I remember a patient once who, in the first stage of consumption, insisted on coming into town each morning from a considerable distance in the country, to look after his business, and to return home again in the afternoon. It mattered not that the sky looked threatening, for he was not afraid of such a trifle, although he knew that the plague spot was in his breast. When expostulated with by friends (and, I am ashamed to say, by myself, for I was ignorant then of the truths I now preach), his reply was, "My brothers and sisters have all died of consumption; they were coddled up, nursed, carried about, confined to bed, and bound in the cords of helplessness by the kindest hands, to the satisfaction of the doctor and of all concerned. But they soon died. I hold the germs of the same disease, and I too shall die; I know it; but my course is different, for I have made up my mind to die in harness; I have kept at my business in resistance to all entreaties, and I am the only one of the family left." The plan adopted by this man was right; he bore the brunt of the disease for months, and, to the best of my knowledge, he is alive, and occupied still.

I recommend every consumptive, whose occupation is in the open air, to take to heart the motto of this man, to make up their minds "to die in harness". They will live the longer for the resolution.

In these remarks I refer only to bodily labour. It is always well to keep the mind easily occupied, but extensive mental exertion or study is quite inadmissible. It leads to muscular inactivity, to seclusion, to an interference indirectly with respiration, to the more rapid evolution of the disease, to death.

Rule vii.—*Cleanliness of body is a special point in the treatment of consumption.* But little need be said to enforce this rule. In health there is always a mutual understanding and a kind of partnership between the skin and lungs. In consumption moderate action of the skin is a relief to the lungs, and as such ought to be encouraged. This is best attained by keeping the skin clean by daily ablution. Let the consumptive boldly take his bath as each morning comes; not a shower bath, not a cold bath, under any impression that water cast on the body in a certain fashion, or at a certain temperature, will give strength, but a tepid cleansing bath, with the temperature from five to ten degrees above that of the body. There is no occasion to stay in the bath a moment longer than to obtain a free ablution; then the patient should rapidly but effectually dry himself all over with a rough towel, and dress with the flannel garment undermost. If oil inunction has been used over night, a little liquid ammonia may be added to the bath water, and a soap will then be made on the body during the ablution.

The clothes of the patient should be kept as clean as possible, and the under clothing should, properly, be changed every second or third day.

Rule viii.—*Marriage of consumptive females for the sake of arresting the course of the disease by pregnancy is morally wrong, and physically mischievous.* In all ranks of life, when young females are the victims of consumption, marriage is sometimes looked to as a means for arresting the disease. There is a general feeling that if a consumptive woman become pregnant, the symptoms of the disease will be at least temporarily suspended. I do not dispute this position, for I have, I believe, witnessed the fact many times of a pregnancy checking the progress of consumption. There are physiological reasons why it should do so. As the blood of the mother goes to the support of the child which she bears, it finds, in the placental structure, and in the lungs of the fœtus, favouring structures for the deposition of tubercular matter. Hence, there is a diversion of the disease, in some measure, from the maternal organs.

But it is because the mother is thus saved at the expense of her offspring that the rule given above should be the more urgently insisted on. These innocents, thus made the scapegoats of their parents' infirmities, come into the world only half mortal; they come into the world to pass through all the miseries of a consumptive life, and, if they survive long enough, to add further misery, in many cases, by propagating other specimens of the half mortal series. This is, and must be considered an infringement of a moral law.

But it is physically wrong also; for what if, through a few months, a life be prolonged? What is the result when the period of pregnancy is past? This, and, without paradox, nothing less; that the end is the quicker for the delay. A few weeks, nay, a few days, and the little half mortal, scapegoat *par excellence,* is left to struggle as it can, onwards, upwards, on the treadwheel of its existence, without the maternal care, or the love that breathes dearest on its own.

Meantime, while yet a temporary respite is sought for, the expectant mother is prevented, in great part, from the performance of that active exercise out of doors which we have seen is so essential in the hygienic treatment of consumption. She thus forfeits what may be a permanent advantage, for one which is temporary, and which goes to perpetuate her own vital deficiencies in her own kith and kin.

Rule ix. *The diet of consumptive patients should be ample, and should contain a larger proportion of the respiratory elements of food than is required in health.* The appetite of consumptive patients is very capricious, and daily grows more so if it is not sharpened up by exercise. When the food taken is not applied to the purposes of nutrition, it is better left untasted; for otherwise it lies undigested in the alimentary canal, and sets up a serious train of dyspeptic symptoms, nausea, and diarrhœa. Kind friends often, with the most provoking and mistaken good nature, thrust upon the consumptive relays of the most improper food, because the necessity for nourishment is so obvious. But the fact is that, when the lungs are acting indifferently, digestion cannot go on actively; since, as Arbuthnot well observed, respiration is "the second digestion". Hence the quantity of food taken by the consumptive person should be small at each meal; but the meals may, if the sensations of the patient require it, be more frequent than in health. Animal food is an absolute necessity, and of all animal foods, mutton is the best. Fatty and oily foods, which constitute the respiratory class, should predominate, and fresh butter, with bread, may be taken almost *ad libitum*, so long as it agrees with the stomach. Cream, too, is very excellent, and the northern luxury of curds and cream is well suited to these cases. Milk, whenever it suits, is advisable as a constant beverage, and good cow's milk, new, answers every purpose; at all events there is, as far as I can gather from cases in which I have seen them tried, no such specific virtues in asses' milk and goats' milk as some have supposed. Tea is nutritious, and may be taken in moderation with perfect safety. Fresh vegetable diets should not be omitted; and fruits, especially roasted apples, are always admissible, except in instances where they excite irregular action of the bowels. The Iceland moss has had a great reputation, as have jellies of different kinds, but these often are slow in digestion, and they have no specific value. Alcoholic drinks in moderate quantities should never be denied the consumptive. Good port wine, unadultered ales, and even brandy and water, are useful. Rum and milk was once a famous remedy, and I believe I have seen it do good, but not uncommonly it gives rise to acidity and flatulency.

In the selection of these various articles of food, the safe plan is to allow the instincts of the patients to guide the practice. These instincts rarely misdirect; but if they are disobeyed, the results are too often disastrous. The one independent rule which should be impressed on the patient by his adviser is that given above, namely, to take in as much of the respiratory foods, especially the fatty and alcoholic foods, as he feels consistent with his desires and with prudence; for as he lives in some measure in a perpetual winter, he, like the Esquimaux, calls the more freely for the supporters of animal combustion. As regards times of eating, let the instinctive feelings again have their way; when hunger calls, let it it be obeyed at whatever season; and when the stomach says "enough", let that order be attended to with equal punctuality.

Rule x.—*The medicinal treatment of consumption should in the main be of the tonic class.* In consumption, the medicines given should be made to assume the characters of food as much as is possible. Cod-liver oil, though used as a medicine, is essentially a food; and in small doses, often repeated (from one to three drachms for a dose), its value is, to my mind, unmistakable. Steel and quinine are invaluable, and, in their way, are also a kind of food. Opium, so absolutely demanded at times to secure rest, has the disadvantage of interfering with nutrition. The four remedies here named, together with gallic acid to meet depressing discharges, supply with me, as a general rule, all the medicinal remedies indicated in uncomplicated cases of pulmonary consumption.

While fully believing in the constitutional origin of consumption, I believe it to be a preventible disorder, and even curable in its early stages; but preventible only, and curable only, by strict attention to hygienic rules. The rules above laid down, however obvious they may seem to professional men, are to the public unknown and unrecognised. For the public expect the remedy in one pill or plaister, and not in a series of instructions tending to bring men to act in obedience with those simple and allwise laws, in which a truly natural state of existence is implied and embodied. If these observations shall tend to impress this important truth on a few minds, they will not have been written in vain.

